# Comparative Phytochemical Profiling and Cellular Antioxidant and Anti-Inflammatory Effects of Wild and Cultivated *Vaccinium* Berries

**DOI:** 10.3390/antiox15070910

**Published:** 2026-07-22

**Authors:** Denisia Pașca, Ionel Fizeșan, Letiția-Elena Mateș, Daniela-Saveta Popa, Ana-Maria Vlase, Oana Mîrza, Lorena Filip, Laurian Vlase, Ioan Tomuță, Lara Manyes, Felicia Loghin

**Affiliations:** 1 Department of Toxicology, Faculty of Pharmacy, “Iuliu Haţieganu” University of Medicine and Pharmacy, 6 Louis Pasteur, 400349 Cluj-Napoca, Romania; denisia.pasca@umfcluj.ro (D.P.); micu.letitia@umfcluj.ro (L.-E.M.); dpopa@umfcluj.ro (D.-S.P.); floghin@umfcluj.ro (F.L.); 2 Biotech AgriFood, Faculty of Pharmacy and Food Sciences, University of Valencia, 46100 Burjassot, Spain; lara.manyes@uv.es; 3 Department of Nutrition and Medical Cosmetics, Faculty of Nursing and Health Sciences, “Iuliu Haţieganu” University of Medicine and Pharmacy, 6 Louis Pasteur, 400349 Cluj-Napoca, Romania; 4 Department of Pharmaceutical Botany, Faculty of Pharmacy, “Iuliu Hațieganu” University of Medicine and Pharmacy, 8 Victor Babes Street, 400012 Cluj-Napoca, Romania; gheldiu.ana@umfcluj.ro; 5 Department of Bromatology, Hygiene, Nutrition, Faculty of Pharmacy, “Iuliu Haţieganu” University of Medicine and Pharmacy, 6 Louis Pasteur, 400349 Cluj-Napoca, Romania; oana.stanciu@umfcluj.ro (O.M.); lfilip@umfcluj.ro (L.F.); 6 Academy of Romanian Scientists (AOSR), 3 Ilfov Street, 050044 Bucharest, Romania; 7 Department of Pharmaceutical Technology and Biopharmacy, Faculty of Pharmacy, “Iuliu Haţieganu” University of Medicine and Pharmacy, 8 Victor Babes Street, 400012 Cluj-Napoca, Romania; laurian.vlase@umfcluj.ro (L.V.); tomutaioan@umfcluj.ro (I.T.)

**Keywords:** *Vaccinium myrtillus*, *Vaccinium corymbosum*, bilberry, blueberry, polyphenols, anthocyanins, antioxidant activity, anti-inflammatory activity, Caco-2 cells, THP-1 cells

## Abstract

Background: *Vaccinium* berries are rich in polyphenolic compounds with recognized health properties; however, studies integrating phytochemical characterization with functional cellular assays remain limited. This study comparatively evaluated wild (*Vaccinium myrtillus* L., VM) and cultivated blueberry (*Vaccinium corymbosum* L., VC) using phytochemical profiling alongside in vitro antioxidant and anti-inflammatory activity testing. Six samples from Romanian regions were extracted using ultrasound-assisted methods, then analyzed for total phenolics, flavonoids, tannins, and antioxidant activity (DPPH, ABTS, FRAP, CUPRAC). Anthocyanins were further characterized by LC–MS/MS. The most bioactive extracts were tested for cytocompatibility, intracellular ROS modulation in Caco-2 cells, and anti-inflammatory activity in LPS-stimulated THP-1 cells. VM extracts showed higher total phenolic, anthocyanin contents, and antioxidant capacity than VC, with LC–MS/MS confirming a richer anthocyanin profile and differences in other phenolic compounds. Both extracts significantly reduced intracellular ROS in H_2_O_2_-stimulated Caco-2 cells and decreased IL-8 secretion in LPS-stimulated THP-1 cells, with a more pronounced dose-dependent anti-inflammatory effect for VM. These findings demonstrate that phytochemical differences between wild and cultivated *Vaccinium* species translate into distinct biological activities at the cellular level. The integrated approach provides a mechanistic link between composition and function, supporting the superior antioxidant and anti-inflammatory potential of VM and its relevance for functional food and nutraceutical applications.

## 1. Introduction

In recent years, berries have attracted considerable scientific attention as dietary sources of bioactive phytochemicals with health-promoting potential. Among the most studied berry genera is *Vaccinium*, a diverse genus within the *Ericaceae* family encompassing approximately 450 species with wide global distribution [1,2,3,4]. Among these, wild bilberries (*Vaccinium myrtillus*, VM) and cultivated blueberries (*Vaccinium corymbosum*, VC) are of particular scientific and nutritional interest. Although both species belong to the same genus, they differ considerably in their botanical characteristics, growing environments, and phytochemical profiles. Bilberries grow naturally in forest ecosystems throughout Europe and constitute a natural source of dietary polyphenols, whereas blueberries are widely cultivated under agronomic conditions and represent an important commercial fruit crop [5,6]. Both fruits are abundant in polyphenols, particularly anthocyanins, which are responsible for their intense blue-purple pigmentation and account for a broad spectrum of biological activities, including antioxidant, anti-inflammatory, antimicrobial, and anticancer effects [7,8,9].

Polyphenols and anthocyanins from *Vaccinium* berries have been extensively investigated for their ability to modulate oxidative stress and inflammatory responses. These compounds scavenge reactive oxygen species (ROS), enhance cellular antioxidant defenses, and interfere with pro-inflammatory signaling pathways. Owing to these properties, berry-derived phytochemicals have attracted considerable interest as dietary agents with potential to prevent or mitigate chronic diseases associated with oxidative stress and inflammation, including cardiovascular and metabolic disorders, as well as neurodegenerative diseases [10,11,12,13].

**Figure 1:**
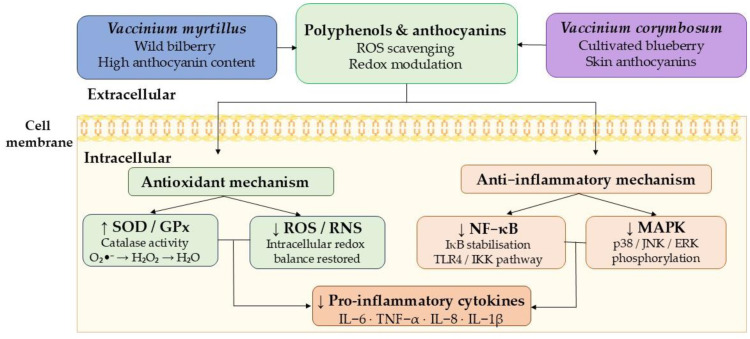
Schematic overview of the antioxidant and anti-inflammatory mechanisms of *Vaccinium* berry polyphenols and anthocyanins in cellular models. Upward arrows (↑) indicate activation or upregulation, whereas downward arrows (↓) indicate inhibition or downregulation.

The principal antioxidant and anti-inflammatory mechanisms of *Vaccinium* polyphenols described in the literature are summarized in Figure 1 [7,12,13,14].

In this context, differences in phytochemical composition between *Vaccinium* species may play an important role in determining their biological effects. Despite belonging to the same genus, wild and cultivated *Vaccinium* species differ substantially in their phytochemical profiles. Wild bilberries are generally reported to contain markedly higher concentrations of anthocyanins, total phenolics, and hydroxycinnamic acids compared to cultivated blueberries [5,15,16,17]. A key structural difference is that bilberries accumulate anthocyanins throughout both the peel and the flesh [16], whereas in cultivated blueberries these pigments are mainly confined to the skin [15]. Recent studies further confirm that wild and cultivated species possess distinct phenolic profiles that enable their differentiation based on anthocyanin and tannin composition, a feature attributed to genetic factors, domestication processes, and agronomic practices [6,15,18]. Although several studies have characterized these compositional differences [15,19], their translation into differential biological effects at the cellular level remains insufficiently explored.

In addition to species-related variability, environmental and agronomic factors critically influence the phytochemical composition of berries. Parameters such as light exposure, temperature, soil composition, altitude, and ripening stage significantly modulate polyphenol biosynthesis, particularly anthocyanin accumulation [6,19,20,21]. Intraspecific variation in anthocyanin composition has been documented in wild bilberry populations across different geographic regions and altitudinal gradients [19,20]. Harvest timing is therefore an important, though underexplored, determinant of antioxidant potential, as later-season berries may accumulate higher levels of phenolic compounds due to prolonged environmental exposure and advanced maturation [6]. However, systematic studies simultaneously assessing the effects of species type and harvest timing on phytochemical composition and in vitro biological activity remain limited.

While the 2,2-diphenyl-1-picrylhydrazyl (DPPH), 2,2′-azinobis-(3-ethylbenzothiazoline-6-sulfonic acid) (ABTS), ferric reducing antioxidant power (FRAP), and cupric ion reducing antioxidant capacity (CUPRAC) assays provide valuable information on radical-scavenging and reducing capacity, they do not fully capture the complexity of biological systems [22]. Increasing attention has therefore been directed toward integrating in vitro cellular models to better assess the physiological relevance of plant-derived phytochemicals. Human-derived cell lines, including intestinal epithelial cells (Caco-2) and immune-derived monocytic cells (THP-1), are widely used to evaluate cytotoxicity, intracellular ROS production, and inflammatory cytokine responses. Caco-2 cells are particularly suitable for investigating the bioactivity of dietary compounds at the intestinal level due to their differentiation into enterocyte-like cells and preserved transport mechanisms [23,24]. THP-1 monocytes are particularly relevant for evaluating anti-inflammatory effects because they retain key innate immune signaling pathways involved in cytokine production following lipopolysaccharide (LPS) stimulation, thereby providing a sensitive model for investigating the immunomodulatory activity of dietary phytochemicals [25,26,27,28]. These complementary models allow for a more biochemically and functionally grounded evaluation of how bioactive compounds interact with key cellular pathways in oxidative stress and inflammation.

This study addresses a significant gap in the current literature by providing a direct comparison between wild and cultivated *Vaccinium* species that integrates phytochemical characterization with functional biological evaluation. Previous research has substantially focused on either compositional profiling or isolated bioactivity assays, with limited efforts to link these aspects within a unified experimental framework. Therefore, the present study aims to combine detailed phytochemical analysis with in vitro assessment of antioxidant and anti-inflammatory effects in relevant cellular models, in order to better understand how species type and environmental factors shape the nutraceutical potential of *Vaccinium* berries. Moreover, by relating compositional variability to biological responses, this approach contributes to bridging the gap between experimental findings and their potential nutritional relevance.

## 2. Materials and Methods

### 2.1. Chemicals and Reagents

Vanillin (99%), sodium carbonate, sodium hydroxide, sodium acetate, potassium chloride, Trolox (6-hydroxy-2,5,7,8-tetramethylchroman-2-carboxylic acid, 97%), 2,2-diphenyl-1-(2,4,6-trinitrophenyl)hydrazine (DPPH), 2,4,6-tris(2-pyridyl)-s-triazine (TPTZ), diammonium 2,2′-azino-bis(3-ethylbenzothiazoline-6-sulfonate) (ABTS), ammonium persulfate, N-acetyl cysteine (NAC), ferric chloride, resazurin, Dulbecco’s modified Eagle’s medium (DMEM), fetal bovine serum (FBS), gentamycin, L-glutamine, GlutaMAX, non-essential amino acids (NEAA), trypsin, 2,7-dichlorofluorescein diacetate (DCFH-DA), lipopolysaccharide (LPS) from *Escherichia coli*, dimethyl sulfoxide (DMSO), and Hanks’ Balanced Salt Solution (HBSS) were purchased from Sigma-Aldrich (Schnelldorf, Germany). Aluminum chloride (AlCl_3_; ≥98%) was obtained from Carl Roth (Karlsruhe, Germany), while Folin–Ciocâlteu reagent, hydrochloric acid (37%), acetic acid, acetone, ethanol, and methanol were sourced from Merck (Darmstadt, Germany). All chemicals used were of analytical grade, and solvents met HPLC standards.

The phenolic reference standards employed for quantification were purchased from Sigma-Aldrich (Steinheim, Germany), Carl Roth (Karlsruhe, Germany), Merck (Darmstadt, Germany), Extrasyntahse (Lyon, France), and Supelco (Bellefonte, PA, USA). For the solvent system, LC-MS grade acetic acid and methanol (Merck KGaA, Darmstadt, Germany) were utilized alongside ultrapure water produced by a Milli-Q Direct 8 system (Millipore, Bedford, MA, USA).

The Caco-2 growth medium Dulbecco’s modified Eagle medium (DMEM) was purchased from Thermo Fisher Scientific (Waltham, MA, USA). Roswell Park Memorial Institute (RPMI) medium for THP-1 cells was obtained from Biowest (Nuaillé, France). Phosphate-buffered saline (PBS) and 4-(2-hydroxyethyl)-1-piperazine-ethanesulfonic acid (HEPES) were sourced from Sigma Chemical Co. (St. Louis, MO, USA).

### 2.2. Plant Material

A total of six samples were harvested between July and September 2023, comprising three bilberry samples (*Vaccinium myrtillus* L.) collected from spontaneous flora and three blueberry samples (*Vaccinium corymbosum* L.) obtained from cultivated sources (Table 1). Plant material was collected from different locations in Alba and Maramureș counties, Romania. All specimens were identified by Dr. Ramona Păltinean (Department of Pharmaceutical Botany, “Iuliu Hațieganu” University of Medicine and Pharmacy, Cluj-Napoca, Romania), and voucher specimens were deposited in the department’s herbarium under the following numbers: VM1 (106.7.2.11), VM2 (106.7.2.2), VM3 (106.7.2.1), VC1 (106.7.3.2), VC2 (106.7.3.3), and VC3 (106.7.3.1). The berries were immediately stored at −20 °C in hermetically sealed plastic containers, protected from light and humidity, until further analysis to preserve their bioactive compounds.

### 2.3. Preparation of Fruit Extracts and Evaluation of pH Influence on Phytochemical Extraction

Fruit matrix extracts were prepared using an ultrasound-assisted extraction (UAE) method adapted from Marzullo et al. [5]. Briefly, 0.6 g of berry sample was mixed with 20 mL of extraction solvent, corresponding to a solid-to-solvent ratio of 0.03 g/mL. The extraction solvent consisted of methanol and Milli-Q water (55:45, *v*/*v*), in accordance with the optimal organic solvent percentage reported by Marzullo et al. [5]. The influence of pH on phytochemical extraction was evaluated by adjusting the solvent to pH = 4 and pH = 7 using hydrochloric acid or sodium hydroxide, as appropriate, as these values were previously identified as optimal for the extraction of total anthocyanins and total phenolic compounds, respectively, from berry matrices using UAE [29]. The extraction was carried out at 35 °C for 20 min. Following extraction, the samples were centrifuged at 3000 rpm for 15 min. The resulting supernatants were filtered through 0.20 µm nylon syringe filters and used for the phytochemical characterization and antioxidant assays.

For the subsequent in vitro biological experiments, fresh extracts were prepared independently from the selected berry samples using the same UAE protocol and the original methanol–water extraction solvent (55:45, *v*/*v*), adjusted to neutral pH. Although both extraction conditions were evaluated during the phytochemical screening, neutral pH extracts were selected for the subsequent analyses because they provided the most appropriate anthocyanin profile for the comparative phytochemical characterization and the following biological experiments. These extracts were subsequently lyophilized using an Advantage 2.0 lyophilizer (SP Scientific, Warminster, PA, USA) for 24 h at −55 °C, followed by an additional 48 h at −25 °C under a pressure of 200 mTorr. The lyophilized extracts were stored at −80 °C, protected from light and moisture, until preparation of stock solutions for the cell culture experiments.

For biological activity assays, lyophilized VM and VC extracts were initially dissolved in DMSO to prepare concentrated stock solutions (200 mg/mL). Stock solutions were subsequently diluted in culture medium to obtain the desired working concentrations for cell exposure. The final DMSO concentration in the culture medium did not exceed 0.2% (*v*/*v*), and equivalent vehicle controls were included in all experiments.

### 2.4. Quantitative Determinations of Total Bioactive Compounds

#### 2.4.1. Total Phenolic Content (TPC)

The TPC of the extracts was quantified using the Folin–Ciocâlteu (FC) spectrophotometric assay according to the method described by Solcan et al. [30]. Briefly, 20 µL of sample was added to a 96-well microplate and mixed with 80 µL of FC reagent diluted 1:10. After 3 min of incubation, 80 µL of 7.5% (*w*/*v*) sodium carbonate solution was added to the mixture. The plate was incubated for 30 min in the dark at room temperature, after which the absorbance was recorded at 760 nm using a Synergy 2 microplate reader (BioTek Instruments, Inc., Winooski, VT, USA), with the reagent blank used as reference. Gallic acid (GA) was employed as the calibration standard (0.01–0.16 mg/mL, r^2^ = 0.9986), and the results were expressed as milligrams of gallic acid equivalents (GAE) per gram of dry weight (DW).

#### 2.4.2. Total Flavonoid Content (TFC)

The TFC of the extracts was determined using a spectrophotometric method performed in 96-well microplates, as previously described [30]. Briefly, 100 µL of extract was mixed with 100 µL of a 2% AlCl_3_ aqueous solution. The reaction mixture was incubated for 15 min at room temperature in the dark. After incubation, the absorbance was recorded at 420 nm against a blank using a microplate reader. Quercetin was used as the calibration standard (0.313–60 µg/mL, R^2^ = 0.9967), and the results were expressed as milligrams of quercetin equivalents (QE) per gram of DW.

#### 2.4.3. Condensed Tannin Content (CTC)

The determination of CTC was conducted through a spectrophotometric approach within 96-well plates. In this method, 50 µL of the extract was combined with 250 µL of a solution containing 0.5% vanillin solution prepared in methanol containing 4% concentrated HCl (*v*/*v*). Subsequently, the plate was placed in darkness and incubated at 30 °C for 20 min, after which the absorbance was measured at 500 nm against a blank, with the same microplate reader. The calibration curve was established using catechin as reference standard (0.01–0.12 mg/mL, R^2^ = 0.9935), and the CTC was expressed as milligrams of catechin equivalents (CE) per gram of DW.

### 2.5. Determination of Antioxidant Activity

The antioxidant activity of the extracts was evaluated using three complementary spectrophotometric assays, including DPPH, ABTS, and FRAP.

#### 2.5.1. DPPH Assay

The DPPH free radical scavenging activity was determined according to a previously described method [30], with minor modifications. Briefly, 10 µL of sample solution was mixed with 90 µL of a 0.1 mM ethanolic DPPH solution in a 96-well microplate. The reaction mixture was incubated for 30 min at room temperature in the dark. Subsequently, the absorbance was measured at 517 nm against a solvent blank using a microplate reader. The radical scavenging activity was calculated using the equation: DPPH scavenging capacity (%) = [(Ac − As)/Ac] × 100, where Ac represents the absorbance of the control and As represents the absorbance of the sample. Trolox was used as the reference standard to construct the calibration curve, and the results were expressed as milligrams of Trolox equivalents (TE) per gram of dry weight (mg TE/g DW).

#### 2.5.2. FRAP Assay

The ferric reducing antioxidant power (FRAP) assay was used to evaluate the reducing capacity of the VM and VC extracts, based on the reduction of the Fe^3+^–TPTZ complex to the blue-colored Fe^2+^–TPTZ form, as previously described by Safta et al. [31]. Briefly, 25 µL of sample solution was mixed with 175 µL of freshly prepared FRAP reagent in a 96-well microplate. The FRAP reagent consisted of 300 mM acetate buffer (pH 3.6), 10 mM TPTZ solution in 40 mM HCl, and 20 mM FeCl_3_·6H_2_O solution in 40 mM HCl, mixed in a volumetric ratio of 10:1:1 (*v*/*v*/*v*). The reaction mixture was incubated for 30 min at room temperature in the dark, and the absorbance was measured at 593 nm using a microplate reader. Trolox was used as the reference standard to construct the calibration curve (0.0031–0.1 mg/mL, R^2^ = 0.9991), and the results were expressed as mg TE/g DW.

#### 2.5.3. ABTS Assay

The antioxidant capacity of the VM and VC extracts was also evaluated using the ABTS radical cation decolorization assay based on the Trolox equivalent antioxidant capacity (TEAC) method, as previously described [32,33,34]. Briefly, 10 µL of sample solution or Trolox standard was mixed with 190 µL of ABTS•^+^ working solution in a 96-well microplate. The ABTS radical cation was generated by reacting ABTS with ammonium persulfate and subsequently diluted with distilled water to obtain an absorbance of approximately 0.70 at 734 nm. The samples were incubated for 30 min in the dark at room temperature, after which the absorbance was recorded at 734 nm using a microplate reader. A Trolox calibration curve was constructed within the concentration range of 0.0031–0.1 mg/mL (R^2^ = 0.9996), and the antioxidant activity was expressed as mg TE/g DW.

#### 2.5.4. CUPRAC Assay

The cupric ion reducing antioxidant capacity (CUPRAC) of the extracts was evaluated using a microplate-based assay adapted from Frumuzachi et al. [35]. The working CUPRAC reagent was freshly prepared by combining 1 M ammonium acetate buffer (pH 7.0), 10 mM CuCl_2_, and 7.5 mM neocuproine solutions at a volumetric ratio of 1:1:0.5. For the assay, 25 μL of each extract was mixed with 175 μL of the freshly prepared reagent in a 96-well microplate. Following incubation in the dark for 30 min at room temperature, absorbance was recorded at 450 nm using a Synergy 2 microplate reader (BioTek Instruments, Inc., Winooski, VT, USA). For the CUPRAC assay, a Trolox calibration curve was constructed over the concentration range of 0.005–0.1 mg/mL (R^2^ = 0.9944), and the antioxidant activity was expressed as mg TE/g DW.

### 2.6. Phytochemical Analysis by Liquid Chromatography–Tandem Mass Spectrometry (LC–MS/MS)

#### 2.6.1. Anthocyanins Analysis

A new LC-MS/MS analytical method was developed for the quantification of 13 anthocyanins in extracts, specifically delphinidin 3-galactoside, delphinidin 3-glucoside, cyanidin-3-O-galactoside, delphinidin 3-rutinoside, cyanidin 3-glucoside, cyanidin 3-arabinoside, cyanidin 3-rutinoside, petunidin 3-glucoside, delphinidin, malvidin 3-glucoside, cyanidin, petunidin, and pelargonidin. The equipment and analytical column were the same as the previously described methods, with the addition of a diode array detector (DAD) for anthocyanin analysis. Chromatographic separation was achieved at 40 °C using a binary gradient of acetonitrile and 0.1% aqueous trifluoroacetic acid (*v*/*v*) at a flow rate of 1 mL/min and an injection volume of 2 μL. The elution profile began at 5% acetonitrile, increasing linearly to 30% over 25 min, followed by a 5-min re-equilibration at 5% acetonitrile. Analytes were monitored in both DAD and MS or MS/MS modes. The DAD was set at 530 nm (20 nm bandwidth) with a reference wavelength of 700 nm. Mass spectrometry detection was performed using an ion trap instrument equipped with an electrospray ionization (ESI) source in positive mode (LC/MSD Ion Trap VL, Agilent, Santa Clara, CA, USA). Optimized source parameters included a capillary voltage of 4000 V, a nebulizer pressure of 60 psi, a drying gas flow of 12 L/min, and a drying gas temperature of 325 °C. The quantification of anthocyanins was made based on DAD signal, after positive qualitative identification based on MS spectra/signal. The limit of detection (LOD) was 0.02 μg/mL and the limit of quantification (LOQ) was 0.1 μg/mL. The calibration curves were found to be linear for all analytes within the concentration range of 0.1–50 μg/mL. Specific retention times, custom MS operating modes, and transitions for each anthocyanin are summarized in the Supplementary Materials (Table S1) [36,37].

#### 2.6.2. Non-Anthocyanin Phenolic Compounds

##### Instrumentation

Chromatographic separations were performed on a Shimadzu Nexera X3 HPLC system (Shimadzu Corporation, Kyoto, Japan) equipped with two LC-40D X3 pumps with an integrated degasser, a SIL-40C X3 autosampler, and a CTO-40S column oven. The liquid chromatograph was coupled to a Shimadzu QTOF 9030 mass spectrometer. Data acquisition and processing were performed using LabSolutions software v. 5.137 (Shimadzu Corporation, Kyoto, Japan).

##### LC-MS/MS Conditions and Method Validation

The analytical methodology for the phenolic compounds was adapted from our previously published protocols [31,36,38,39]. Chromatographic separation was achieved on an Agilent Zorbax SB-C18 column (100 mm × 3.0 mm i.d., 3.5 µm particle size) (Agilent Technologies, Santa Clara, CA, USA) maintained at 48 °C. The mobile phase consisted of 0.2% (*v*/*v*) acetic acid in water (Solvent A) and 0.2% (*v*/*v*) acetic acid in methanol (Solvent B). Gradient elution was applied as follows: 0–30 min, a linear gradient from 5% to 70% B, followed by a 4-min re-equilibration step at 5% B. The flow rate was 1 mL/min, and the injection volume was 1 µL. Mass spectrometry detection was performed in negative ionization mode using either high-resolution MS (HR-MS) or high-resolution MS/MS (HR-MS/MS), both maintaining a mass accuracy tolerance of 15 ppm. The ESI source parameters were source voltage, −2000 V; interface temperature, 300 °C; nebulizing gas flow, 3 L/min; heating gas flow (air), 15 L/min; drying gas flow (nitrogen), 15 L/min; desolvation line temperature, 300 °C; and heat block temperature, 400 °C.

The QTOF mass spectrometer was operated using multiple scheduled time events for both qualitative and quantitative analyses.

For qualitative analysis, an HR-MS scan was scheduled between 0.8 and 30 min alongside Data-Dependent Acquisition (DDA) over the same timeframe. Spectral data obtained from the DDA event were utilized for the qualitative confirmation of compounds (requiring a spectral match score > 90 and an ion ratio calculation for two spectral qualifier ions within a ±20% tolerance of the library spectra). For positive library matches, peak areas were integrated from the HR-MS data, and relative compound concentrations were expressed as equivalents of the most structurally similar active moiety [40,41].

Quantitative analysis was conducted using a specific time-scheduled event for each available analytical standard (starting 0.8 min before and ending 0.8 min after its retention time) in HR-MS or HR-MS/MS mode. A compound was quantified only after positive qualitative identification via DDA spectral data [40,41]. The quantitative method was validated for linearity, limit of detection (LOD), limit of quantification (LOQ), precision, and accuracy. Linearity was assessed across the 0.05–50 µg/mL range (R^2^ ≥ 0.998). LOD and LOQ were defined at signal-to-noise (S/N) ratios of ≥10 and ≥20, respectively. The method yielded strong intra-day precision (≤4.8% RSD), inter-day precision (≤7.2% RSD), and accuracy (92.4–104.7% recovery). Full calibration parameters are detailed in Supplementary Table S1 [40,41].

To guarantee the high mass accuracy of the spectral data (<3 ppm mass error), an internal standard calibration step using a 400 mg/L sodium iodide solution (P/N 220-91239-90; Shimadzu Corporation, Kyoto, Japan) was run during each analysis between 0.1 and 0.3 min (prior to the chromatographic dead time). Mass corrections were automatically applied to the spectral data by the LabSolutions software.

Quantification relied on monitoring the pseudo-molecular ion (SIM, MS) or specific fragmentation transitions (MRM, MS/MS); transition parameters are outlined in Supplementary Table S1 and in Brudiu et al. [41]. Results were expressed as micrograms of analyte per mL of vegetal extract (μg/mL).

#### 2.6.3. Phytochemical Data Interpretation and Analysis

Data acquisition and subsequent processing were managed using ChemStation (vB01.03) and DataAnalysis (v5.3) software packages (Agilent Technologies, Santa Clara, CA, USA). To ensure rigorous compound authentication, a multi-parameter validation approach was implemented. This included aligning experimental retention times (Rt) with analytical standards, matching UV spectra, and confirming diagnostic MS and MS/MS fragmentation patterns, focusing on both precursor and corresponding product ions. Across all employed methodologies, the quantitative evaluation of the identified secondary metabolites was performed utilizing external calibration curves, as previously detailed [31,36,38,39].

### 2.7. In Vitro Biological Activity on Cell Lines

#### 2.7.1. Cell Culture

Human colon adenocarcinoma Caco-2 cells (ATCC HTB-37) were obtained from CLS Cell Lines Service GmbH (Eppelheim, Germany). The cells were cultured as adherent monolayers in 75 cm^2^ polystyrene tissue-culture flasks at a seeding density of 25,000 cells/cm^2^. Cells were maintained in DMEM, 4.5 g/L glucose supplemented with 10% FBS, 1% HEPES, 1% NEAA, and 1% penicillin–streptomycin. The cultures were subcultured or used for experiments once they reached approximately 70–80% confluence.

THP-1 human monocytic cells (ATCC TIB-202) were purchased from the American Type Culture Collection (ATCC, Manassas, VA, USA). The cells were maintained in RPMI-1640 medium supplemented with 10% FBS and 1% penicillin–streptomycin. THP-1 monocytes were used directly without differentiation, as preliminary experiments confirmed adequate cytokine responses to LPS stimulation in the undifferentiated state. Cell cultures were incubated at 37 °C in a humidified atmosphere containing 5% CO_2_ and were routinely maintained at a density of 2–8 × 10^5^ cells/mL, with the culture medium renewed every two days.

#### 2.7.2. Preparation of Extract Solutions

Stock solutions of 200 mg/mL in DMSO were prepared from each lyophilized plant extract. The stock solutions were further diluted in DMSO to obtain working solutions of 5, 12.5, 25, 37.5, 50, 75, 100, 150, and 200 mg/mL. Subsequently, the working solutions were used to prepare the final solutions with concentrations ranging from 20 to 800 µg/mL in cell culture media. All extract solutions were sterilized by filtration through 0.22 µm syringe filters before addition to cell cultures.

#### 2.7.3. Viability Assays

Cell viability following extract exposure was evaluated using the Alamar Blue (resazurin) assay on Caco-2 and THP-1 cells, as previously described [30]. Caco-2 and THP-1 cells between passages 20–40 were seeded in 96-well plates at densities of 60,000 and 30,000 cells/well, respectively, in 100 µL of culture medium and allowed to adhere or stabilize, respectively, for 24 h before exposure. Subsequently, 100 µL of extract-containing medium was added to each well, resulting in a final volume of 200 µL/well. After incubation with the extracts, cells were exposed to 200 µM resazurin for 2–3 h. Fluorescence intensity was measured using a Synergy 2 Multi-Mode Microplate Reader (Agilent BioTek, Santa Clara, CA, USA) (λ__excitation_ = 530/25 nm; λ__emission_ = 590/35 nm). All experiments were performed in three independent biological replicates, each including six technical replicates. A negative control consisting of cells treated with culture medium containing 0.2% DMSO was included in all assays. Cell viability was expressed relative to the negative control, which was set at 100%.

#### 2.7.4. Intracellular ROS Measurement

The antioxidant capacity of the VM and VC extracts at the cellular level was evaluated in Caco-2 cells using the ROS-sensitive probe DCFH-DA, as previously described [30]. Caco-2 cells were selected due to their preserved membrane transport mechanisms, making them a suitable in vitro model for investigating the antioxidant activity of dietary phenolic compounds. Cells were treated for 24 h with non-cytotoxic concentrations of the extracts (200, 400, and 800 µg/mL). After exposure, the cells were washed with PBS and incubated with 50 µM DCFH-DA in HBSS for 2 h. Following removal of excess dye, the cells were further exposed for 2 h to either 250 µM H_2_O_2_ to induce oxidative stress (stimulated condition) or HBSS alone (basal condition). The oxidation of DCFH to the fluorescent product dichlorofluorescein (DCF) was monitored using a Synergy 2 Multi-Mode Microplate Reader (Agilent BioTek, Santa Clara, CA, USA) (λ_excitation = 485/20 nm; λ_emission = 528/20 nm). NAC (5 mM) was used as a reference antioxidant.

#### 2.7.5. Anti-Inflammatory Activity Assessment by Cytokine Quantification

The anti-inflammatory activity of the VM and VC extracts was evaluated by measuring the secretion of pro-inflammatory cytokines in cell culture supernatants using enzyme-linked immunosorbent assay (ELISA). Preliminary experiments were conducted in Caco-2, and THP-1 cells stimulated with LPS; however, only THP-1 cells exhibited a robust and reproducible cytokine response under the selected experimental conditions, supporting their suitability as an immune-relevant model for anti-inflammatory evaluation. Therefore, subsequent analyses were performed using this cell line.

THP-1 cells were stimulated with 0.1 µg/mL LPS from *Escherichia coli* (Sigma-Aldrich, Steinheim, Germany) and simultaneously treated with four selected non-cytotoxic concentrations of the extracts (600, 400, 200, and 100 µg/mL) for 24 h. Before evaluating anti-inflammatory activity, cell viability was confirmed to exclude potential cytotoxic effects of the extracts under LPS stimulation. Following treatment, the culture supernatants were collected and stored at −70 °C until further analysis.

The concentrations of IL-1β, IL-6, IL-8, and TNF-α were determined using commercial ELISA kits (FineTest Biotech Inc., Wuhan, China) according to the manufacturer’s instructions.

### 2.8. Statistical Analysis

All experiments were performed in triplicate (*n* = 3), and the results are expressed as mean ± standard deviation (SD). Statistical analyses were conducted using SigmaPlot 11.0 software (Systat Software Inc., San Jose, CA, USA). Data distribution and homogeneity of variances were evaluated using the Shapiro–Wilk test. Differences between groups were assessed using Student’s *t*-test or one-way analysis of variance (ANOVA), followed by Holm–Šídák multiple comparison post hoc tests when appropriate. A *p*-value ≤ 0.05 was considered statistically significant.

## 3. Results

### 3.1. Phytochemical Characterization and Antioxidant Activity

To investigate the phytochemical characteristics of the analyzed berries, all six samples (three *Vaccinium myrtillus* and three *Vaccinium corymbosum*) were initially evaluated under both neutral and acidic extraction conditions. Phytochemical composition and antioxidant capacity were assessed to identify the samples with the highest bioactive potential. Based on these results, one representative sample from each species (VM3 and VC3) was selected for the subsequent in vitro biological experiments.

Considerable variation in phytochemical composition was observed among samples, with extraction pH markedly influencing TPC, TFC, and CTC values (Table 2). Acidic extraction significantly increased TPC in both VM and VC samples. In VM extracts, TPC increased from 25.65–47.21 mg/g under neutral conditions to 28.63–48.95 mg/g under acidic conditions, corresponding to an overall increase of approximately 5–12%, with the largest proportional increase observed for VM1. Conversely, TFC generally decreased by approximately 15–30% following acidic extraction, while CTC exhibited a more pronounced reduction, decreasing by up to approximately 40%.

A similar pattern was observed for VC extracts. TPC increased from 15.61–27.25 mg/g at neutral pH to 19.80–27.70 mg/g under acidic conditions, whereas both TFC and CTC decreased under acidic conditions. Overall, VC samples consistently exhibited lower TPC than VM samples, while changes in TFC and CTC followed the same pH-dependent trends observed for the wild bilberry extracts. Significant differences among samples and extraction conditions were confirmed by one-way ANOVA followed by Holm–Šídák multiple comparison test (Table 2).

Two-way ANOVA revealed that TPC was significantly influenced by species, whereas extraction pH had no significant effect. In contrast, TFC and CTC were not significantly affected by species but were significantly influenced by extraction pH, with neutral pH resulting in higher extraction yields than acidic pH.

The antioxidant activity of all VM and VC extracts was further evaluated using four complementary assays (DPPH, ABTS, FRAP, and CUPRAC), revealing consistent species- and pH-dependent differences (Table 3). Across all assays, VM extracts generally exhibited higher antioxidant activity than VC extracts. Acidic extraction increased DPPH radical scavenging activity in VM samples by approximately 6–18%, with the highest value recorded for VM3 (144.37 mg TE/g). A similar variation was observed for VC; however, the antioxidant activities were consistently lower than those of VM.

Unlike DPPH, ABTS activity was only slightly affected by extraction pH in both species, with comparable values observed under neutral and acidic conditions. FRAP values generally decreased after acidic extraction, particularly in VM samples, indicating a reduced ferric ion reducing capacity under these conditions. A similar decline was observed for VC extracts. CUPRAC values followed the same overall tendency, with neutral extracts generally exhibiting higher cupric ion reducing capacity than the corresponding acidic extracts, although the magnitude of the decrease varied among samples. Significant differences among samples and extraction conditions were confirmed by one-way ANOVA followed by Holm–Šídák multiple comparison test (Table 3).

Post hoc multiple comparison analysis identified statistically significant differences among the 12 extract groups for all antioxidant capacity assays (DPPH, ABTS, FRAP, and CUPRAC) (*p* < 0.05). Pearson’s correlation analysis revealed strong positive correlations between TPC and all antioxidant assays, namely DPPH (r = 0.869), ABTS (r = 0.783), FRAP (r = 0.760), and CUPRAC (r = 0.890), indicating that the antioxidant capacity of the extracts was primarily associated with their total phenolic content. In contrast, TFC exhibited only weak to moderate correlations with the antioxidant assays, while no significant correlations were observed for CTC.

The targeted LC-MS analysis revealed marked qualitative and quantitative differences in anthocyanin composition between VM and VC extracts (Table 4). Overall, VM extracts contained higher concentrations of all quantified anthocyanins than VC extracts and exhibited a more complex anthocyanin profile. Among the VM samples, VM3 consistently displayed the highest concentrations for most individual anthocyanins, particularly cyanidin-3-O-galactoside, delphinidin-3-glucoside, and cyanidin-3-glucoside.

In contrast, VC extracts contained considerably lower anthocyanin levels, with several compounds remaining below the detection limit. Delphinidin-3-galactoside and cyanidin-3-O-galactoside were detected in all VC samples, whereas delphinidin-3-glucoside, cyanidin-3-glucoside, petunidin-3-glucoside, and malvidin-3-glucoside were absent or detected only sporadically. Extraction pH influenced the abundance of individual anthocyanins in a compound-dependent manner, with no uniform trend observed across all analytes.

Comprehensive LC-MS/MS profiling identified a broad range of non-anthocyanin phenolic compounds belonging to four major classes, namely hydroxybenzoic acids and derivatives, hydroxycinnamic acids and derivatives, flavanols, and flavonols and their derivatives (Table 5). Distinct qualitative and quantitative differences were observed between the VM3 and VC3 extracts.

Among hydroxybenzoic acids and their derivatives, VM3 contained higher concentrations of gallic acid, protocatechuic acid, gallic acid O-glucoside, and protocatechuic acid-3-O-glucoside, whereas VC3 was characterized by higher levels of several glycosylated derivatives, including 4-O-methylgallic acid-3-O-glucoside, 6-O-galloyl-β-D-glucose, galloyl-glucose sulfate, and vanillic acid-4-O-glucoside. Syringic acid was detected exclusively in VC3, while vanillic acid was not detected in either extract.

Hydroxycinnamic acid derivatives were dominated by chlorogenic acid, which was present at higher concentrations in VC3 (1110.07 ± 85.47 µg/g DW) than in VM3 (708.50 ± 41.10 µg/g DW). Likewise, 4-O-caffeoylquinic acid was detected only in VC3, whereas p-coumaric acid O-glucoside was identified exclusively in VM3. Caffeic acid was detected in both extracts at comparable concentrations.

The flavanol fraction also differed between the two species. VM3 was characterized by higher concentrations of epicatechin and several procyanidins, including procyanidins B2, B4, B5, and C1, whereas VC3 contained higher amounts of catechin and procyanidin B1. Procyanidin A2 was detected only in VM3.

Marked differences were also observed in the flavonol profile. Hyperoside, isoquercitrin, quercitrin, rutin, and several kaempferol and isorhamnetin glycosides were more abundant or exclusively detected in VC3. In contrast, VM3 contained higher concentrations of myricetin and its glycosylated derivatives, including myricetin-3-O-glucoside, which was not detected in VC3.

Overall, the LC-MS/MS analysis demonstrated distinct phytochemical fingerprints for the two *Vaccinium* species, with differences observed not only in the relative abundance of individual phenolic compounds but also in the occurrence of several species-specific metabolites across the identified phenolic classes. A comprehensive overview of the LC-MS/MS profiles obtained for all analyzed berry samples (VM1–VM3 and VC1–VC3) is presented in Supplementary Table S2.

### 3.2. In Vitro Biological Activity: Cytocompatibility, Antioxidant, and Anti-Inflammatory Effects

Human intestinal epithelial Caco-2 cells and THP-1 monocytes were both employed to evaluate cell viability after treatment exposure, with both cell lines additionally subjected to LPS stimulation to examine their responsiveness. Intracellular ROS production was specifically investigated in Caco-2 cells as a model of intestinal oxidative stress. Although both cell lines were exposed to inflammatory challenge conditions, only THP-1 monocytes demonstrated a significant inflammatory response, validating their use as the primary model for anti-inflammatory assessment. Consequently, the modulation of cytokine secretion was evaluated exclusively in THP-1 cells.

#### 3.2.1. Cell Viability

**Figure 2:**
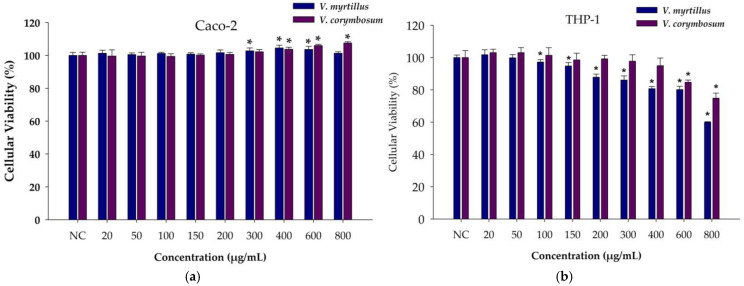
Cytotoxicity of *Vaccinium myrtillus* and *Vaccinium corymbosum* extracts evaluated using the Alamar Blue assay on the (**a**) Caco-2 intestinal cell line and (**b**) THP-1 immune cells after 24 h exposure. Results are expressed as mean ± standard deviation (SD) of three biological replicates (each including six technical replicates). Values are presented relative to the negative control (NC) (100%). Asterisks (*) indicate statistically significant differences compared to the negative control (ANOVA followed by Holm–Sidak post hoc test, *p* < 0.05).

Cell viability was assessed in Caco-2 and THP-1 cell lines following exposure to increasing concentrations (20–800 µg/mL) of VM3 and VC3 extracts (Figure 2).

In Caco-2 cells, both extracts maintained cell viability close to or above 100% across all tested concentrations. A slight but statistically significant increase in viability was observed at higher concentrations (≥300 µg/mL), particularly for VC3, which reached the highest values at 800 µg/mL.

In THP-1 cells, both extracts maintained high cell viability at lower and moderate concentrations (20–100 µg/mL). Although a concentration-dependent decrease was observed at higher doses, particularly at ≥400 µg/mL, viability remained above ~60% even at the highest tested concentration, suggesting moderate cytotoxic effects. VM3 produced a slightly greater reduction in viability compared to VC3.

Overall, both *Vaccinium* extracts preserved high cell viability in Caco-2 cells across all tested concentrations. In THP-1 cells, both extracts showed a similar dose-dependent response, with noticeable decreases in viability only at higher concentrations, highlighting a cell type-dependent effect.

#### 3.2.2. Antioxidant Capacity

**Figure 3:**
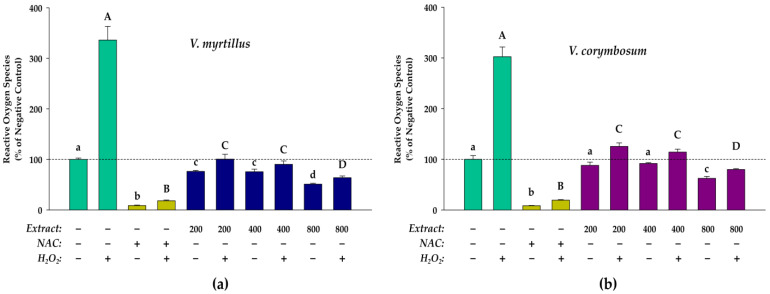
Intracellular reactive oxygen species (ROS) levels of (**a**) *Vaccinium myrtillus* and (**b**) *Vaccinium corymbosum* extracts evaluated using the DCFH-DA assay on the Caco-2 intestinal cell line. Cells were incubated with the extracts (200, 400, and 800 µg/mL) or NAC (5 mM) for 24 h and subsequently exposed to DCFH-DA. The antioxidant potential was assessed under stimulated and non-stimulated conditions following a 2 h exposure in the presence or absence of H_2_O_2_. Data are presented as relative means ± standard deviation (SD) of three biological replicates and expressed as percentages relative to the negative control (100%). Different letters (a–d for non-stimulated conditions and A–D for stimulated conditions) indicate statistically significant differences between groups; groups labeled with the same letter are not significantly different (ANOVA followed by Holm–Sidak post hoc test, *p* < 0.05).

Intracellular ROS levels were quantified in Caco-2 cells treated with VM3 and VC3 extracts (200–800 µg/mL) under both basal conditions and following H_2_O_2_-induced oxidative stress (Figure 3).

Exposure to H_2_O_2_ (250 µM) significantly increased ROS production compared to the negative control in both experimental sets. Treatment with N-acetylcysteine (NAC, 5 mM) markedly reduced ROS levels, confirming the responsiveness of the assay.

For VM3, all tested concentrations led to a reduction in intracellular ROS compared to the H_2_O_2_-stimulated condition. The most pronounced decrease was observed at 800 µg/mL, followed by 400 µg/mL and 200 µg/mL. Under basal conditions, VM-treated cells exhibited ROS levels comparable to or slightly lower than the negative control.

Similarly, VC3 extracts significantly attenuated ROS generation in H_2_O_2_-treated cells. The reduction was concentration-dependent, with the highest effect observed at 800 µg/mL. At 200 and 400 µg/mL, VC also significantly decreased ROS levels compared to the positive control, although to a lesser extent than the highest concentration. Under non-stimulated conditions, VC treatments maintained ROS levels close to baseline.

Both extracts demonstrated a capacity to reduce intracellular ROS levels in Caco-2 cells, with statistically significant differences between treated groups and the H_2_O_2_ control, as indicated by distinct lettering (*p* < 0.05).

#### 3.2.3. Anti-Inflammatory Capacity

Stimulation of THP-1 cells with LPS (0.1 µg/mL) resulted in a noticeable increase in IL-8 secretion compared to the unstimulated control. Specifically, IL-8 levels increased from approximately 75 pg/mL in control cells to around 100 pg/mL following LPS exposure, confirming the induction of an inflammatory response. Among the cytokines evaluated (IL-1β, IL-6, IL-8, and TNF-α), only IL-8 showed detectable modulation under the experimental conditions, whereas IL-1β, IL-6, and TNF-α did not exhibit significant changes following LPS stimulation or extract exposure.

**Figure 4:**
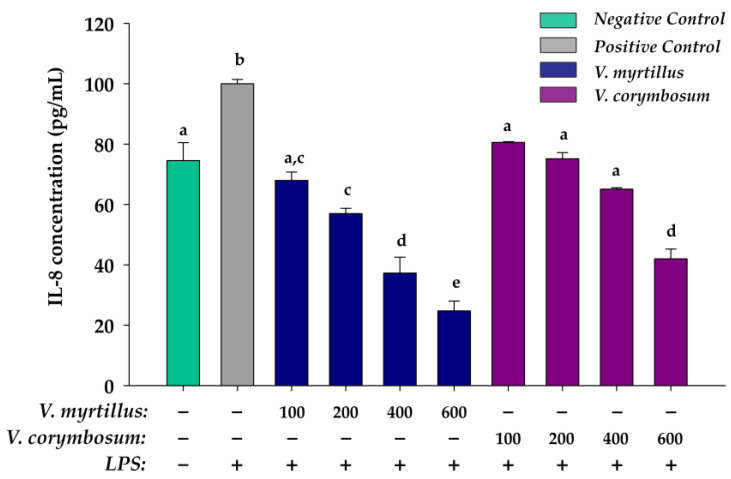
Effect of *Vaccinium myrtillus* and *Vaccinium corymbosum* extracts on IL-8 production under LPS-induced inflammatory conditions. Cells were exposed for 24 h to increasing concentrations (100, 200, 400, and 600 μg/mL) of each extract in the presence of LPS. IL-8 levels were quantified in the cell culture supernatants and are presented as mean ± standard deviation (*n* = 3). Different letters indicate statistically significant differences between groups (ANOVA followed by Holm–Sidak post hoc test, *p* < 0.05); groups sharing the same letter are not significantly different.

Exposure to VM3 extract significantly reduced IL-8 secretion in a concentration-dependent manner under LPS stimulation. Relative to the LPS control, IL-8 levels were reduced by approximately 32%, 43%, 63%, and 75% following treatment with 100, 200, 400, and 600 µg/mL VM3 extract, respectively, with the highest concentration exhibiting the strongest inhibitory effect. Statistically significant differences between concentrations were indicated by distinct superscript letters (Figure 4).

Likewise, VC3 extract also reduced IL-8 secretion in LPS-stimulated THP-1 cells, although the effect was less pronounced compared to VM3 at equivalent concentrations. At 100 and 200 µg/mL, IL-8 levels remained relatively high (80 and 75 pg/mL, respectively), not significantly different from each other. A moderate reduction was observed at 400 µg/mL (65 pg/mL), while a more substantial decrease occurred at 600 µg/mL (42 pg/mL), which was significantly lower than the LPS control.

Both extracts demonstrated anti-inflammatory activity by attenuating LPS-induced IL-8 secretion, with VM3 exhibiting a stronger and more consistent dose-dependent effect compared to VC3.

## 4. Discussion

The present study comparatively evaluated *Vaccinium myrtillus* and *Vaccinium corymbosum* fruits by integrating phytochemical characterization with functional cellular assays. The results demonstrate that differences in phenolic composition between wild and cultivated species are associated with distinct antioxidant and anti-inflammatory effects at the cellular level, highlighting the functional relevance of their phytochemical profiles.

### 4.1. Phytochemical Profile of the Extracts

Despite belonging to the same genus, wild and cultivated *Vaccinium* species differed substantially in their phytochemical composition. Bilberries are generally characterized by higher concentrations of total phenolics, anthocyanins, flavonols, and hydroxycinnamic acids compared to cultivated blueberries [42]. In addition, structural differences exist in pigment distribution: bilberries contain anthocyanins throughout both the peel and pulp, whereas cultivated blueberries primarily accumulate these compounds in the skin [15]. Recent metabolomic and chromatographic analyses further confirm that wild and cultivated berries exhibit distinct phenolic profiles, enabling their differentiation based on anthocyanin and tannin composition. These compositional differences are largely attributed to genetic factors, domestication processes, and agronomic practices, which can significantly influence the biosynthesis and accumulation of secondary metabolites [43].

The present study reveals notable variability in the phytochemical profile and antioxidant activity of both VM and VC extracts, influenced by sample geographic origin (VM1–VM3; VC1–VC3) and extraction pH conditions. Such intraspecific variability in phenolic composition and antioxidant capacity is well documented for wild bilberry populations and is generally attributed to differences in environmental stressors, altitude, soil properties, and harvest timing [6,19]. A recent study by Mujanović et al. confirmed that environmental factors significantly shape polyphenol content and anthocyanin composition across wild bilberry populations, reinforcing the importance of geographic and seasonal context in phytochemical characterization [19,20].

TPC was consistently higher under acidic extraction conditions across all samples. In aqueous solution, anthocyanins exist in a pH-dependent equilibrium between structural forms; at pH values of approximately 1–2, the flavylium cation predominates and exhibits high water solubility and strong UV-Vis absorbance, while at higher pH values conversion to colorless hemiketal and chalcone forms reduces their detectability [44]. Although anthocyanins constitute a major fraction of total phenolics in *Vaccinium* species, the higher TPC values observed under acidic extraction conditions likely reflect enhanced recovery of the overall phenolic fraction rather than anthocyanins alone, as LC-MS analysis demonstrated that several individual anthocyanins were present at higher concentrations in the neutral extracts [5,45].

Conversely, both TFC and CTC decreased under acidic extraction conditions. The reduction in flavonoid content may be explained by the differential acid stability of individual flavonoid subclasses, which depends on their hydroxylation pattern and glycosylation state. Polyhydroxylated flavonoids such as myricetin and quercetin derivatives are particularly susceptible to structural modifications during acidic extraction [46]. Likewise, the marked decrease in CTC can be attributed to the analytical characteristics of the vanillin assay, which primarily detects free flavan-3-ols and terminal units of proanthocyanidin polymers. Acid-catalyzed depolymerization alters proanthocyanidin chain length and reduces the availability of reactive terminal units, thereby affecting the measured tannin content [47]. These findings highlight that extraction pH differentially influences distinct phenolic subclasses and demonstrate that total phenolic content alone does not fully describe extract composition.

The antioxidant assays further demonstrated that extraction pH differentially affected the antioxidant mechanisms measured by each analytical method. Acidic extracts consistently exhibited higher DPPH radical-scavenging activity, in agreement with their higher TPC values. Anthocyanins and other phenolic compounds are effective hydrogen atom and electron donors, and their radical-scavenging capacity is closely associated with the number and position of hydroxyl substituents, which stabilize the resulting semiquinone radicals [48]. In contrast, ABTS values were only minimally affected by extraction pH, suggesting that the overall electron-transfer capacity of the extracts remained relatively stable despite compositional changes. Conversely, both FRAP and CUPRAC generally decreased following acidic extraction, indicating that reducing capacity toward ferric and cupric ions depends not only on total phenolic concentration but also on the preservation of specific phenolic subclasses, particularly flavonoids and condensed tannins. Together, these complementary assays demonstrate that antioxidant activity cannot be adequately described by a single analytical method, as different assays probe distinct reaction mechanisms and are influenced differently by extract composition.

Our findings are in agreement with those reported by Bilgin et al. [49], who also observed cultivar-dependent variation in antioxidant activity of *Vaccinium corymbosum* using DPPH and FRAP assays and demonstrated a close relationship between antioxidant capacity and phenolic composition. The inclusion of DPPH, ABTS, FRAP, and CUPRAC assays in the present study provides a broader assessment of the chemical antioxidant potential of the extracts by evaluating both radical-scavenging and reducing mechanisms. Nevertheless, future studies incorporating additional aqueous radical systems, such as PTIO•, would further improve the mechanistic characterization of *Vaccinium* phytochemicals.

Despite the higher TPC and DPPH values observed under acidic extraction conditions, neutral pH extracts were selected for the subsequent biological experiments. Neutral extraction better preserves the native structural diversity of flavonoids, condensed tannins, and anthocyanins, as reflected by the higher TFC and CTC values and the more representative individual anthocyanin profiles obtained by LC-MS. Furthermore, neutral extraction conditions more closely resemble the physicochemical environment in which these compounds are present in foods and are subsequently subjected to gastrointestinal digestion, thereby providing extracts that are more representative for in vitro biological evaluation and future bioavailability studies [50].

Across all assessed parameters, VM samples consistently exhibited higher levels of total phenolics, anthocyanins, and antioxidant activity compared to VC. This is consistent with the findings of Hellström et al. [15], who reported substantially higher concentrations of phenolics and anthocyanins in wild bilberries than in cultivated blueberries. Similarly, a recent comprehensive review concluded that wild VM generally contains greater amounts of total phenolics, anthocyanins, and hydroxycinnamic acids than cultivated VC, contributing to its superior antioxidant and anti-inflammatory potential [6,21].

The expanded LC-MS/MS anthocyanin profiling further confirmed the distinct phytochemical characteristics of the two species. Wild bilberry extracts consistently contained substantially higher concentrations of all major anthocyanins than cultivated blueberry extracts, with cyanidin-3-O-galactoside and delphinidin-3-glucoside among the predominant compounds. In contrast, several anthocyanins were absent or detected only at trace levels in VC samples, reflecting the simpler anthocyanin composition of cultivated blueberries. These findings are consistent with the well-established anatomical distribution of anthocyanins in the two species, as anthocyanins accumulate throughout both the peel and pulp of *V. myrtillus*, whereas in *V. corymbosum* they are largely confined to the fruit skin [6,15,42]. Moreover, the greater exposure of wild bilberries to environmental stressors, including higher UV radiation, lower temperatures, and nutrient-poor soils, is known to stimulate phenylpropanoid metabolism and anthocyanin biosynthesis, further contributing to their enhanced pigment accumulation [1,15].

The variability observed among the analyzed samples may also be partially associated with differences in harvest timing. Notably, the VM3 and VC3 samples, collected later in the harvesting season (September), exhibited the highest levels of phenolic compounds, anthocyanins, and antioxidant activity among the investigated extracts. These findings are consistent with previous reports indicating that polyphenol accumulation increases during fruit ripening and that environmental conditions throughout berry development contribute to the final phytochemical composition of *Vaccinium* fruits [20,51]. Consequently, the extracts selected for subsequent biological assays originated from samples harvested during a favorable maturation stage, which may have contributed to their superior phytochemical profile and biological activity.

Beyond anthocyanins, the comprehensive HRMS profiling revealed clear differences in the non-anthocyanin phenolic composition of the two *Vaccinium* species. Although chlorogenic acid was the predominant hydroxycinnamic acid in both extracts, it was present at considerably higher concentrations in *V. corymbosum*. Conversely, *V. myrtillus* was characterized by higher levels of several hydroxybenzoic acid derivatives, including gallic acid, protocatechuic acid, and their glycosylated forms, together with a flavanol-rich profile comprising elevated concentrations of epicatechin and multiple procyanidins (B2, B4, B5, and C1). These compounds are recognized for their strong radical-scavenging, metal-chelating, and anti-inflammatory properties and likely contribute to the superior antioxidant performance consistently observed for the VM extracts [10,42,52]. In contrast, VC exhibited a flavonol-rich profile characterized by higher concentrations of hyperoside, isoquercitrin, quercitrin, rutin, and several kaempferol- and isorhamnetin-derived glycosides. Flavonol glycosides have also been associated with antioxidant, anti-inflammatory, and cytoprotective activities, suggesting that cultivated blueberries possess a distinct, yet biologically relevant, phenolic composition. Collectively, these findings demonstrate that the phytochemical differences between the two species extend beyond anthocyanins and involve multiple phenolic subclasses, supporting the concept that wild bilberries are characterized by greater overall phenolic richness, whereas cultivated blueberries display a complementary phytochemical profile dominated by flavonol glycosides and hydroxycinnamic acids [6,10,42].

### 4.2. Biological Activity and Mechanistic Insights

Both VM and VC extracts significantly reduced intracellular ROS levels in H_2_O_2_-stimulated Caco-2 cells in a concentration-dependent manner. These results are in line with a broad body of evidence showing that polyphenol-rich berry extracts protect intestinal cell models against oxidative stress by attenuating ROS production and activating endogenous antioxidant defense pathways [53]. Specifically, a comprehensive review by Felgus-Lavefve et al. [23] confirmed that blueberry phytochemicals modulate cellular mechanisms of oxidative stress through downregulation of the NF-κB pathway and reduction in ROS and lipid peroxidation in cell models. More recently, a 2025 metabolomics study using an intestinal cell model (IEC-6 cells) demonstrated that VM extract significantly inhibited H_2_O_2_-induced intracellular ROS production and nitrosative stress, corroborating the protective antioxidant role of bilberry polyphenols at the intestinal level [43]. Supporting this, a recent study using wild blueberry extract in Caco-2 cells demonstrated that anthocyanins and chlorogenic acids attenuate TNF-α-induced oxidative stress and improve intestinal barrier integrity, further highlighting the cytoprotective potential of *Vaccinium*-derived polyphenols in intestinal epithelial models [54].

The protective antioxidant effect is attributed not only to direct radical scavenging by polyphenols but also to the upregulation of endogenous enzymatic defenses, including superoxide dismutase (SOD), catalase (CAT), and glutathione peroxidase (GPx) [10,55]. These mechanisms are consistent with activation of the Nrf2/ARE pathway, a key cellular antioxidant response axis that is known to be induced by dietary flavonoids and anthocyanins [56]. Notably, proanthocyanidins, which are particularly abundant in VM as confirmed by our LC–MS data, have been shown to activate the Nrf2/ARE pathway in intestinal epithelial cells specifically by inhibiting ubiquitin-mediated Nrf2 degradation, thereby promoting the antioxidant response [57]. The concentration-dependent ROS reduction observed in our study supports the view that higher loads of bioactive compounds translate into greater intracellular antioxidant efficacy, and that the superior effect of VM at equivalent concentrations reflects its richer supply of anthocyanins, procyanidins, and phenolic acids [10,23].

The choice of Caco-2 cells as the experimental model for assessing intracellular antioxidant activity is well-justified. These cells represent a widely used in vitro model for investigating the effects of dietary polyphenols on intestinal oxidative stress [58]. Importantly, both extracts maintained high cell viability in Caco-2 cells across all tested concentrations, indicating that the observed ROS reduction is a genuine antioxidant response rather than a cytotoxic artifact (Figure 2).

It is worth noting that while anthocyanins are predominantly associated with antioxidant effects in normal epithelial cells, context-dependent pro-oxidant activity has been reported in cancer cell lines, where ROS accumulation may contribute to antiproliferative or apoptotic effects [25,59]. In this context, the marked concentration-dependent reduction in THP-1 cell viability observed with VM extract at higher concentrations (≥800 µg/mL) may reflect a cell-type-specific cytotoxic response, consistent with reports of selective antiproliferative activity of VM extracts against transformed and immune-derived cell lines, but not against normal epithelial cells [60].

In the present study, conducted in non-malignant Caco-2 cells under oxidative stress induced by H_2_O_2_, the predominant response was cytoprotective ROS reduction, consistent with the anticipated dietary antioxidant role of these compounds [59]. Overall, these findings reinforce the value of *Vaccinium* berry extracts as dietary antioxidants capable of mitigating oxidative stress in intestinal epithelial cells, with VM demonstrating a comparatively stronger effect, attributable to its superior phenolic content and diversity [10,15,23,43].

Among the cytokines evaluated (IL-1β, IL-6, IL-8, and TNF-α), only IL-8 showed detectable modulation under the experimental conditions. Therefore, only IL-8 was selected for quantitative analysis and is presented below. Both VM and VC extracts significantly reduced IL-8 secretion in LPS-stimulated THP-1 monocytes, revealing in vitro anti-inflammatory activity. IL-8, a key CXC chemokine with potent neutrophil-recruiting activity, is a well-established marker of pro-inflammatory activation in monocytic cells and was induced approximately 1.3-fold by LPS stimulation in this study. Notably, VM extract produced a more pronounced and consistent dose-dependent inhibition of IL-8, reaching the greatest reduction at the highest tested concentration (600 μg/mL), while VC showed a more moderate effect, particularly at lower concentrations (Figure 4).

The superior anti-inflammatory effect of VM is consistent with prior studies highlighting the potent immunomodulatory properties of bilberry extracts. Triebel et al. [25] demonstrated that a VM extract and individual anthocyanins modulated inflammatory gene expression in intestinal cell models, including suppression of IL-8 secretion, an effect that was dependent on the structural stability of anthocyanins under cell culture conditions. Furthermore, Bayazid et al. [61] showed that bilberry anthocyanin fractions exerted strong antioxidant and anti-inflammatory effects, with significant suppression of pro-inflammatory mediators attributed to the inhibition of NF-κB and MAPK signaling pathways. The NF-κB pathway, activated downstream of TLR4 by LPS, drives the transcription of chemokines including IL-8, TNF-α, IL-1β, and IL-6, as well as COX-2 and iNOS. Anthocyanins are thought to interfere with this pathway at multiple levels, including prevention of IκBα degradation, inhibition of p65 nuclear translocation, and suppression of NF-κB-dependent gene transcription [12,13,62].

The anti-inflammatory activity of VC extract was also evident, although less pronounced than that of VM. Falcone et al. [26] reported that an anthocyanin-enriched VC extract exerted antiproliferative and anti-inflammatory activities in vitro and ex vivo, including modulation of inflammatory cytokine expression in relevant cell models. Samad et al. [63] similarly demonstrated that Korean blueberry extracts inhibited nitric oxide production and attenuated inflammatory responses in macrophage models. The comparatively lower efficacy of VC observed in our study may be primarily associated with its lower total phenolic content, together with differences in anthocyanin concentration and overall phenolic composition relative to VM, as the biological activity of berry extracts is strongly influenced by their phytochemical profile [7,61,64].

The clear dose–response relationship observed for both extracts is consistent with published data showing that phenolic-rich berry extracts exert stronger anti-inflammatory effects at higher concentrations through modulation of inflammatory signaling pathways and transcription factors [13,61,65]. The interplay between oxidative stress and inflammatory signaling is well established, as ROS can activate pathways such as NF-κB and promote cytokine production [10,13,55,56]. The reduction in IL-8 observed in THP-1 cells may therefore reflect a combination of antioxidant effects and direct modulation of inflammatory signaling pathways by bilberry polyphenols [66].

In the present study, only IL-8 showed detectable modulation by the extracts under LPS stimulation, whereas IL-1β, IL-6, and TNF-α were not significantly altered. This selective cytokine response may be related to differences in cytokine expression kinetics in THP-1 cells, as IL-8 is often induced more rapidly following inflammatory stimulation, while cytokines such as IL-6 and TNF-α may require different activation conditions, longer incubation periods, or a more differentiated cellular phenotype to exhibit pronounced changes [67,68,69]. The absence of significant modulation of these cytokines may therefore reflect the specific experimental conditions employed in the present study, including the 24 h exposure period and the use of non-differentiated THP-1 cells, rather than a complete lack of inflammatory responsiveness.

These results confirm that both *Vaccinium* species possess meaningful in vitro anti-inflammatory activity in an LPS-stimulated monocytic model, with VM emerging as the more potent anti-inflammatory agent under the tested conditions, potentially due to its higher anthocyanin content and broader, more diverse phenolic composition. These outcomes support the potential application of bilberry- and blueberry-derived extracts as functional dietary ingredients or nutraceutical components for the management of oxidative stress and low-grade inflammation [3,6,9,13,61,70].

The findings of the present study demonstrate that differences in phytochemical composition between wild VM and cultivated VC are directly associated with distinct antioxidant and anti-inflammatory responses at the cellular level. The higher polyphenolic and anthocyanin content observed in VM was consistently reflected in its superior capacity to reduce intracellular ROS and modulate IL-8 secretion. These results highlight the importance of considering both species type and harvest-related variability when evaluating the biological potential of berry-derived compounds.

These findings are consistent with previous in vivo studies demonstrating the anti-inflammatory potential of *Vaccinium*-derived extracts. Anthocyanin-rich *V. myrtillus* extracts have been shown to attenuate experimentally induced inflammation in mice, whereas *V. corymbosum* extracts reduced inflammatory edema and associated inflammatory markers in rodent models [13,71]. Together, these preclinical findings support the potential of *Vaccinium* polyphenols as functional food ingredients capable of modulating inflammatory processes beyond cell culture models, while reinforcing the biological relevance of the present in vitro observations.

Nevertheless, although the present findings are supported by previous preclinical evidence, they were obtained under controlled in vitro conditions and should therefore be interpreted with caution. Factors such as bioavailability, metabolism, food matrix interactions, and realistic dietary intake may substantially influence the biological activity of berry-derived polyphenols in vivo. The concentration-dependent responses observed in the present study suggest that their anti-inflammatory efficacy is closely linked to effective cellular exposure, highlighting the need for further animal and human studies to establish physiologically achievable doses and confirm their translational relevance.

Another limitation of the present study is that detailed environmental and climatic parameters (e.g., altitude, temperature, precipitation, and sunlight exposure) were not systematically recorded for the berry collection sites. These factors are recognized determinants of the phytochemical composition of *Vaccinium* species and may have contributed to the variability observed among samples. Nevertheless, the primary objective of this study was to compare the phytochemical composition and biological activity of wild and cultivated berries in order to identify the most suitable matrix for subsequent mechanistic investigations. Future studies integrating comprehensive environmental metadata with phytochemical and biological profiling would provide a more comprehensive understanding of the environmental factors governing the composition and bioactivity of *Vaccinium* berries.

## 5. Conclusions

This study demonstrates that VM and VC are not interchangeable in terms of phytochemical composition and biological activity. The higher content and structural diversity of phytochemicals in VM were associated with enhanced antioxidant and anti-inflammatory effects, as evidenced by reduced intracellular ROS levels in Caco-2 cells and decreased IL-8 secretion in LPS-stimulated THP-1 cells.

Both extracts exhibited high cytocompatibility and significant bioactivity; however, VM consistently showed a stronger, concentration-dependent effect. These findings highlight the importance of polyphenolic composition as a determinant of functional efficacy, as well as the influence of extraction conditions on phytochemical recovery.

Taking all the results into account, VM emerges as a particularly promising source of bioactive compounds for functional food and nutraceutical applications. Further studies are needed to elucidate the underlying molecular mechanisms and to confirm the bioavailability and physiological relevance of these effects in vivo.

## Figures and Tables

**Table 1:** Harvesting locations and collection details of the selected species.

Harvested Species	Location	Date	GPS Position
*V. myrtillus* L.–1 (VM 1)	Mătișești, Alba county, Romania	29 July 2023	46°30′54″ N 22°53′41″ E
*V. myrtillus* L.–2 (VM 2)	Munții Maramureșului, Maramureș county	16 August 2023	47°46′58″ N 24°33′8″ E
*V. myrtillus* L.–3 (VM 3)	Cavnic, Maramureș county, Romania	12 September 2023	47°40′18″ N 23°50′33″ E
*V. corymbosum* L.–1 (VC 1)	Cavnic, Maramureș county, Romania	25 August 2023	47°39′37″ N 23°53′51″ E
*V. corymbosum* L.–2 (VC 2)	Cavnic, Maramureș county, Romania	12 September 2023	47°41′17″ N 23°54′19″ E
*V. corymbosum* L.–3 (VC 3)	Sat Șugatag, Maramureș county, Romania	24 September 2023	47°48′13″ N 23°54′17″ E

**Table 2:** Total phenolic (TPC), flavonoid (TFC), and tannin (CTC) of *Vaccinium myrtillus* (VM1-3) and *Vaccinium corymbosum* (VC1-3) extracts under neutral and acidic pH conditions (mg/g DW).

		TPC	TFC	CTC
VM1	Neutral pH	25.65 ± 1.25 ^a^	1.98 ± 0.20 ^a^	31.54 ± 1.18 ^a^
VM2		33.91 ± 0.05 ^b^	1.61 ± 0.03 ^b^	24.12 ± 1.63 ^b,c^
VM3		47.21 ± 0.53 ^c^	1.88 ± 0.07 ^a^	24.95 ± 1.63 ^b,c,d^
VM1	Acidic pH	28.63 ± 0.97 ^d^	1.53 ± 0.05 ^b^	22.65 ± 1.03 ^b^
VM2		36.30 ± 0.01 ^e^	1.12 ± 0.02 ^c^	14.61 ± 0.30 ^e^
VM3		48.95 ± 1.10 ^c^	1.49 ± 0.05 ^b,d^	15.97 ± 0.15 ^e^
VC1	Neutral pH	19.16 ± 0.39 ^f^	1.55 ± 0.01 ^b^	27.04 ± 0.15 ^d^
VC2		15.61 ± 0.50 ^g^	0.84 ± 0.04 ^e^	15.02 ± 0.30 ^e^
VC3		27.25 ± 0.36 ^a,d^	2.79 ± 0.01 ^f^	35.82 ± 1.03 ^f^
VC1	Acidic pH	20.29 ± 0.60 ^f^	1.31 ± 0.01 ^c,d^	26.10 ± 0.89 ^c,d^
VC2		19.80 ± 0.65 ^f^	0.85 ± 0.01 ^e^	13.46 ± 0.15 ^e^
VC3		27.70 ± 0.27 ^d^	1.85 ± 0.02 ^a^	24.64 ± 0.89 ^b,c,d^

TPC—total phenolic content (mg GAE/g); TFC—total flavonoid content (mg QE/g); CTC—condensed tannin content (mg CE/g). Data are expressed as mean ± SD (*n* = 3). Different superscript letters indicate statistically significant differences between groups (one-way ANOVA followed by Holm–Šídák multiple comparison test, *p* < 0.05).

**Table 3:** Antioxidant activity of *Vaccinium myrtillus* (VM1-3) and *Vaccinium corymbosum* (VC1-3) extracts under neutral and acidic pH extraction conditions determined by DPPH, ABTS, FRAP, and CUPRAC assays (mg TE/g DW).

		DPPH	ABTS	FRAP	CUPRAC
VM1	Neutral pH	110.87 ± 1.00 ^a^	75.32 ± 4.44 ^a^	109.72 ± 3.52 ^a,b^	39.15 ± 1.02 ^a,b^
VM2		101.87 ± 2.97 ^b^	70.96 ± 3.58 ^a^	79.11 ± 5.85 ^c^	42.60 ± 1.49 ^b,c^
VM3		135.63 ± 0.50 ^c^	81.21 ± 6.70 ^a^	112.93 ± 7.03 ^b^	48.48 ± 1.30 ^d^
VM1	Acidic pH	130.77 ± 2.47 ^c^	74.33 ± 3.95 ^a^	82.02 ± 3.51 ^c^	37.66 ± 0.79 ^a,e,f^
VM2		116.10 ± 3.47 ^a^	69.46 ± 2.95 ^a^	63.28 ± 0.65 ^d^	39.84 ± 1.85 ^a,b^
VM3		144.37 ± 1.00 ^d^	74.90 ± 3.03 ^a^	96.60 ± 6.72 ^a^	44.12 ± 0.58 ^c^
VC1	Neutral pH	62.77 ± 2.97 ^e^	38.62 ± 3.57 ^b^	36.62 ± 6.25 ^e^	34.58 ± 1.90 ^f,g^
VC2		32.77 ± 1.97 ^f^	22.31 ± 2.39 ^c,d^	22.98 ± 1.54 ^f^	35.03 ± 1.40 ^e,f,g^
VC3		100.07 ± 0.50 ^b^	41.40 ± 5.50 ^b^	65.25 ± 5.38 ^d^	38.44 ± 0.27 ^a,e^
VC1	Acidic pH	75.67 ± 2.47 ^g^	32.05 ± 4.69 ^b,d^	24.96 ± 2.43 ^e,f^	31.78 ± 1.00 ^g^
VC2		55.10 ± 1.00 ^h^	18.55 ± 3.17 ^c^	13.82 ± 2.78 ^f^	32.59 ± 1.56 ^g^
VC3		97.97 ± 2.47 ^b^	35.08 ± 3.69 ^b^	37.04 ± 3.61 ^e^	36.48 ± 1.00 ^a,e,f^

DPPH—antioxidant activity (mg TE/g); ABTS—antioxidant activity (mg TE/g); FRAP—antioxidant activity (mg TE/g); CUPRAC—antioxidant activity (mg TE/g). Data are expressed as mean ± SD (*n* = 3). Different superscript letters indicate statistically significant differences between groups (one-way ANOVA followed by Holm–Šídák multiple comparison test, *p* < 0.05).

**Table 4:** Anthocyanin content (mg/g DW) of *Vaccinium myrtillus* (VM1–VM3) and *Vaccinium corymbosum* extracts (VC1–VC3) under neutral and acidic pH conditions.

		D-3-gal	D-3-glu	C-3-O-gal	C-3-glu	C-3-ara	P-3-glu	M-3-glu
VM1	Neutral pH	0.421 ± 0.013 ^a^	0.691 ± 0.028 ^a^	0.492 ± 0.015 ^a^	0.508 ± 0.030 ^a^	0.291 ± 0.015 ^a^	0.398 ± 0.004 ^a^	0.504 ± 0.005 ^a^
VM2		1.081 ± 0.022 ^b^	1.900 ± 0.038 ^b^	1.121 ± 0.034 ^b^	1.116 ± 0.045 ^b,c^	0.753 ± 0.023 ^b^	1.039 ± 0.042 ^b^	1.285 ± 0.039 ^b^
VM3		1.225 ± 0.037 ^c^	2.061 ± 0.062 ^b^	2.604 ± 0.078 ^c^	1.233 ± 0.062 ^c,d^	0.892 ± 0.018 ^c^	1.130 ± 0.057 ^c^	1.254 ± 0.025 ^b^
VM1	Acidic pH	0.431 ± 0.017 ^a^	0.691 ± 0.014 ^a^	0.537 ± 0.027 ^a^	0.522 ± 0.031 ^a^	0.260 ± 0.008 ^d^	0.383 ± 0.011 ^a^	0.489 ± 0.039 ^a^
VM2		0.967 ± 0.068 ^d^	1.642 ± 0.099 ^c^	1.106 ± 0.022 ^b^	1.102 ± 0.011 ^b^	0.722 ± 0.007 ^e^	0.932 ± 0.047 ^d^	1.147 ± 0.023 ^c^
VM3		1.194 ± 0.084 ^c^	1.980 ± 0.059 ^b^	1.376 ± 0.041 ^d^	1.305 ± 0.091 ^d^	0.877 ± 0.018 ^c^	1.069 ± 0.032 ^b,c^	1.300 ± 0.052 ^b^
VC1	Neutral pH	0.102 ± 0.006 ^e^	ND	0.043 ± 0.003 ^e^	ND	0.015 ± 0.001 ^f^	ND	ND
VC2		0.174 ± 0.002 ^e,f,g^	ND	0.088 ± 0.005 ^e^	ND	0.044 ± 0.001 ^f^	ND	ND
VC3		0.174 ± 0.003 ^e,f,g^	ND	0.058 ± 0.003 ^e^	ND	0.029 ± 0.0003 ^f^	0.169 ± 0.003 ^e^	ND
VC1	Acidic pH	0.194 ± 0.008 ^f,g^	ND	0.086 ± 0.004 ^e^	ND	0.030 ± 0.001 ^f^	ND	ND
VC2		0.163 ± 0.003 ^e,f^	0.015 ± 0.0001 ^c^	0.073 ± 0.002 ^e^	ND	0.044 ± 0.003 ^f^	ND	ND
VC3		0.215 ± 0.006 ^g^	ND	0.058 ± 0.001 ^e^	ND	0.030 ± 0.002 ^f^	0.489 ± 0.020 ^f^	ND

D-3-gal—Delphinidin 3-galactoside, D-3-glu—Delphinidin 3-glucoside, C-3-O-gal—Cyanidin-3-O-galactoside, C-3-glu—Cyanidin 3-glucoside, C-3-ara—Cyanidin 3-arabinoside, P-3-glu—Petunidin 3-glucoside, M-3-glu—Malvidin 3-glucoside; Values are expressed as mean ± standard deviation (mg/g DW). ND = Not detected (<limit of detection of the analytical method). Different superscript letters indicate statistically significant differences between groups (one-way ANOVA followed by Holm–Šídák multiple comparison test, *p* < 0.05).

**Table 5:** Identification and Quantification of Bioactive Compounds in *Vaccinium myrtillus* (VM3) and *Vaccinium corymbosum* (VC3) by LC-MS/MS expressed as μg/g DW.

Category	Compound	Concentration	
		VM3	VC3
Hydroxybenzoic acids and derivatives	Gallic acid	16.833 ± 1.067 ^a^	3.700 ± 0.133 ^b^
	Protocatechuic acid	38.933 ± 1.900 ^a^	1.933 ± 0.067 ^b^
	Vanillic acid	ND	ND
	Syringic acid	3.067 ± 0.133 ^a^	10.167 ± 0.300 ^b^
	Gallic acid O-galactoside	ND	0.767 ± 0.033
	Gallic acid O-glucoside	7.167 ± 0.367 ^a^	3.900 ± 0.133 ^b^
	3-O-Methylgallic acid	2.333 ± 0.067 ^a^	0.633 ± 0.033 ^b^
	3-O-Methylgallic acid 4-O-glucoside	1.967 ± 0.067 ^a^	2.467 ± 0.133 ^b^
	4-O-Methylgallic acid 3-O-glucoside	6.433 ± 0.300 ^a^	16.533 ± 0.933 ^b^
	6-O-galloyl-β-D-glucose	1.867 ± 0.033 ^a^	14.467 ± 0.767 ^b^
	Galloyl-glucose sulfate	ND	43.933 ± 2.800
	Protocatechuic acid-3-O-glucoside	17.467 ± 0.667 ^a^	11.100 ± 0.767 ^b^
	Vanillic acid-4-O-glucoside	10.533 ± 0.733 ^a^	2.567 ± 0.067 ^b^
	Salicylic acid O-glucoside	0.867 ± 0.033	ND
Hydroxycinnamic acids and derivatives	1-O-Caffeoyl-β-D-glucose	ND	ND
	3-O-Caffeoyl-β-D-glucose	ND	1.367 ± 0.100
	Chlorogenic acid	708.500 ± 41.100 ^a^	1110.067 ± 85.467 ^b^
	4-O-Caffeoylquinic acid	ND	18.033 ± 0.767
	3,5-Dicaffeoylquinic acid	ND	6.200 ± 0.233
	Caffeic acid	2.800 ± 0.100 ^a^	3.567 ± 0.200 ^b^
	p-Coumaric acid O-glucoside	1.400 ± 0.100	ND
	p-Coumaric acid	1.533 ± 0.100	ND
	trans-Ferulic acid-4-O-glucoside	8.500 ± 0.567 ^a^	3.267 ± 0.167 ^b^
	Ferulic acid	ND	ND
Flavanols	Gallocatechin	17.633 ± 1.400 ^a^	1.767 ± 0.067 ^b^
	Catechin	1.133 ± 0.067 ^a^	2.054 ± 0.012 ^b^
	Epicatechin	53.267 ± 1.800 ^a^	3.767 ± 0.133 ^b^
	Procyanidin A2	2.700 ± 0.133	ND
	Procyanidin B1	6.233 ± 0.267 ^a^	18.367 ± 0.567 ^b^
	Procyanidin B2	90.867 ± 6.467 ^a^	6.833 ± 0.533 ^b^
	Procyanidin B4	15.067 ± 0.700	ND
	Procyanidin B5	6.433 ± 0.167	ND
	Procyanidin C1	41.433 ± 1.900 ^a^	5.167 ± 0.400 ^b^
Flavonols and derivatives	Hyperoside	477.700 ± 37.267 ^a^	307.267 ± 10.433 ^b^
	Isoquercitrin	55.667 ± 2.733 ^a^	131.767 ± 9.767 ^b^
	Quercetin-3-O-arabinoside	22.800 ± 1.633 ^a^	24.533 ± 1.167 ^a^
	Quercetin-3-O-robinobioside	ND	1.667 ± 0.067
	Quercetin-3-O-glucose-6-acetate	ND	4.767 ± 0.100
	Quercitrin	28.667 ± 1.133 ^a^	618.367 ± 19.800 ^b^
	Rutin	ND	132.233 ± 7.933
	Myricetin	6.233 ± 0.467	ND
	Myricetin-3-O-galactoside	46.367 ± 1.433 ^a^	43.833 ± 2.633 ^a^
	Myricetin-3-O-glucoside	44.400 ± 2.800	ND
	Kaempferol-3-O-galactoside	0.900 ± 0.033	ND
	Kaempferol-3-O-glucoside	ND	9.300 ± 0.300
	Kaempferol-3-O-rutinoside	ND	2.000 ± 0.067
	Isorhamnetin-3-O-glucoside	4.633 ± 0.167	ND
	Isorhamnetin-3-O-rutinoside	ND	10.967 ± 0.567
	Syringetin-3-O-galactoside	27.133 ± 0.667 ^a^	20.567 ± 1.333 ^b^

Values are expressed as mean ± standard deviation (μg/g DW). ND = Not detected (<limit of detection of the analytical method). Different superscript letters indicate statistically significant differences between VM3 and VC3 (one-way ANOVA followed by Holm–Šídák multiple comparison test, *p* < 0.05).

## Data Availability

Data are contained in the article.

## Supplementary Materials

The following supporting information can be downloaded at: https://www.mdpi.com/article/10.3390/antiox15070910/s1, Table S1: LC-MS/MS Analytical Parameters and Calibration Data for Anthocyanins Analysis; Table S2: Targeted LC-MS Quantification of Phenolic Compounds in Wild and Cultivated *Vaccinium* spp. Berries (µg/g DW).

## Abbreviations

The following abbreviations are used in this manuscript:

ABTS	2,2-azino-bis(3-ethylbenzothiazoline-6-sulfonic acid)
ANOVA	Analysis of variance
ATCC	American Type Culture Collection
CAT	Catalase
COX-2	Cyclooxygenase-2
CTC	Condensed tannin content
CXCL8	C-X-C motif chemokine ligand 8 (IL-8 gene)
DCF	Dichlorofluorescein
DCFH-DA	2,7-dichlorofluorescein diacetate
DMEM	Dulbecco’s Modified Eagle’s Medium
DMSO	Dimethyl sulfoxide
DPPH	2,2-diphenyl-1-(2,4,6-trinitrophenyl)hydrazine
DW	Dry weight
ELISA	Enzyme-linked immunosorbent assay
FBS	Fetal bovine serum
FC	Folin–Ciocâlteu
FRAP	Ferric reducing antioxidant power
GA	Gallic acid
GAE	Gallic acid equivalents
GPx	Glutathione peroxidase
HBSS	Hanks’ Balanced Salt Solution
HEPES	4-(2-hydroxyethyl)-1-piperazine-ethanesulfonic acid
HPLC	High-performance liquid chromatography
IκBα	Inhibitor of NF-κB alpha
IL-1β, IL-6, IL-8	Interleukins 1β, 6, and 8
iNOS	Inducible nitric oxide synthase
LC–MS	Liquid chromatography–mass spectrometry
LPS	Lipopolysaccharide
MAPK	Mitogen-activated protein kinase
NAC	N-acetylcysteine
NC	Negative control
NEAA	Non-essential amino acids
NF-κB	Nuclear factor kappa B
Nrf2/ARE	Nuclear factor erythroid 2-related factor 2/Antioxidant response element
PBS	Phosphate-buffered saline
QE	Quercetin equivalents
ROS	Reactive oxygen species
RPMI	Roswell Park Memorial Institute (medium)
SD	Standard deviation
SOD	Superoxide dismutase
TE	Trolox equivalents
TEAC	Trolox equivalent antioxidant capacity
TFC	Total flavonoid content
THP-1	Human monocytic cell line (ATCC TIB-202)
TLR4	Toll-like receptor 4
TNF-α	Tumor necrosis factor alpha
TPC	Total phenolic content
TPTZ	2,4,6-tris(2-pyridyl)-s-triazine
UAE	Ultrasound-assisted extraction
VC/VC1–VC3	*Vaccinium corymbosum* samples
VM/VM1–VM3	*Vaccinium myrtillus* samples
