# Supplementary material for: Comparative Phytochemical Profiling and Cellular Antioxidant and Anti-Inflammatory Effects of Wild and Cultivated Vaccinium Berries
Source: Antioxidants (Basel). 2026 Jul 22;15(7):910. doi: 10.3390/antiox15070910 (PMC13405888; doi:10.3390/antiox15070910)
Supplement: Supplementary file 1 [file antioxidants-15-00910-s001.zip › antioxidants-4396422-supplementary.pdf]

Table S1. LC-MS/MS Analytical Parameters and Calibration Data for Anthocyanins Analysis

No.	Anthocyanin	Retention time (min)	MS operating mode	Precursor ion *m/z*	Product ion *m/z*	Calibration curve slope	Calibration curve intercept	Correlation coefficient	Inter-day (n=5) precision at LLOQ, %	Inter-day (n=5) accuracy at LLOQ
1	Delphinidin 3-galactoside	7.2	MS/MS (SRM)	465.3	303.2	3.2341	0.1441	0.9950	6.5	8.2
2	Delphinidin 3-glucoside	8.1	MS/MS (SRM)	465.3	303.2	2.0687	0.0937	0.9920	10.3	9.2
3	Cyanidin-3-O-galactoside	8.8	MS/MS (SRM)	449.3	287.1	2.2260	0.1322	0.9901	10.6	-1.1
4	Delphinidin 3-rutinoside	8.9	MS/MS (SRM)	611.5	303.2	1.5078	0.1829	0.9980	7.0	3.4
5	Cyanidin 3-glucoside	9.8	MS/MS (SRM)	449.3	287.1	2.2990	-0.0063	0.9999	5.9	8.7
6	Cyanidin 3-arabinoside	10.6	MS/MS (SRM)	419.3	287.1	2.1626	0.1325	0.9970	8.1	-2.7
7	Cyanidin 3-rutinoside	10.7	MS/MS (SRM)	595.5	287.1	1.7795	0.1336	0.9999	5.5	-4.8
8	Petunidin 3-glucoside	11	MS/MS (SRM)	479.4	317.2	2.1844	-0.0667	0.9941	7.1	4.0
9	Delphinidin	12.3	MS (SIM)	303.2	303.2	4.5500	-0.2500	0.9927	5.2	8.5
10	Malvidin 3-glucoside	13.8	MS/MS (SRM)	493.4	331.2	2.1772	0.0927	0.9990	7.2	-0.7
11	Cyanidin	15.4	MS (SIM)	287.2	287.2	3.7778	-0.1667	0.9943	4.7	8.0
12	Petunidin	16.6	MS (SIM)	317.2	317.2	4.3556	0.1667	0.9972	11.5	-1.0
13	Pelargonidin	18.3	MS (SIM)	271.2	271.2	3.0111	0.1333	0.9982	9.6	7.1

SRM- single reaction monitoring, SIM- single ion monitoring, LLOQ – lower limit of quantification

Table S2. Targeted LC-MS Quantification of Phenolic Compounds in Wild and Cultivated *Vaccinium* spp. Berries (µg/g DW)

**Compound**	**VM1A**	**VM1N**	**VM2A**	**VM2N**	**VM3A**	**VC1A**	**VC1N**	**VC2A**	**VC2N**	**VC3A**
Gallic acid O-galactoside	ND	ND	ND	ND	ND	ND	ND	ND	ND	0.700±0.033
3-O-Methylgallic acid 4-O-glucoside	1.033±0.033	1.367±0.100	0.433±0.033	0.433±0.033	1.967±0.100	4.967±0.333	5.100±0.233	4.733±0.200	4.967±0.300	2.333±0.133
Gallic acid O-glucoside	3.733±0.067	5.267±0.167	1.933±0.067	2.100±0.133	7.100±0.367	9.800±0.767	9.667±0.233	4.633±0.267	4.967±0.267	3.533±0.167
3-O Methylgallic acid	0.600±0.033	2.800±0.067	0.733±0.033	0.867±0.067	2.333±0.067	1.133±0.033	1.267±0.100	0.633±0.033	0.767±0.067	0.433±0.033
4-O-Methylgallic acid 3-O-glucoside	4.133±0.100	5.200±0.200	2.233±0.100	2.133±0.167	6.800±0.267	18.500±0.967	18.600±1.200	12.900±0.633	13.367±0.733	15.967±0.767
Protocatechuic acid-3-O-glucoside	4.267±0.100	5.367±0.133	2.067±0.133	2.033±0.133	18.000±0.433	5.567±0.300	5.667±0.267	7.767±0.500	8.400±0.400	10.067±0.200
6-O-galloyl-beta-D-glucose	1.333±0.067	1.600±0.067	0.633±0.033	0.700±0.033	1.867±0.100	1.767±0.033	1.800±0.133	35.600±2.200	38.433±2.067	13.700±0.267
Galloyl-glucose sulfate	ND	ND	ND	ND	ND	7.567±0.500	8.100±0.400	4.567±0.233	5.267±0.367	40.233±2.300
Vanillic acid-4-O-glucoside	9.267±0.367	10.867±0.267	8.733±0.500	9.067±0.367	10.333±0.400	4.733±0.167	4.933±0.200	3.633±0.100	3.867±0.167	2.433±0.067
3-O-Caffeoyl-beta-D-glucose	ND	ND	ND	ND	ND	1.200±0.100	1.033±0.067	4.933±0.133	0.400±0.000	1.233±0.067
p-Coumaric acid-O-glucoside	1.767±0.067	2.000±0.100	0.967±0.067	0.800±0.067	1.500±0.033	ND	ND	ND	ND	ND
Salicylic acid O-glucoside	1.133±0.033	1.333±0.033	1.300±0.100	1.300±0.033	0.900±0.033	ND	ND	ND	ND	ND
1-O-Caffeoyl-beta-D-glucose	ND	ND	ND	ND	ND	2.933±0.100	2.400±0.067	ND	ND	ND
trans-Ferulic acid-4-O-glucoside	8.967±0.700	10.000±0.367	5.367±0.167	5.067±0.367	8.933±0.333	8.967±0.467	8.900±0.267	7.200±0.467	7.533±0.167	2.433±0.067
Procyanidin B5	8.733±0.533	9.167±0.633	8.433±0.667	8.033±0.600	7.133±0.433	ND	ND	ND	ND	ND
3,5-Dicaffeoylquinic acid	ND	ND	ND	ND	ND	ND	ND	4.600±0.133	5.400±0.433	4.700±0.100
Quercetin-3-O-arabinoside	13.000±0.600	14.267±0.933	9.033±0.667	9.933±0.300	21.533±1.733	16.567±0.900	22.067±1.133	7.267±0.200	10.567±0.333	16.700±0.633
Kaempferol-3-O-galactoside	ND	ND	ND	ND	0.767±0.067	ND	ND	ND	ND	ND
Kaempferol-3-O-rutinoside	ND	ND	ND	ND	ND	ND	ND	ND	ND	1.533±0.067
Isorhamnetin-3-O-glucoside	5.533±0.133	6.233±0.500	3.700±0.133	4.033±0.200	4.633±0.300	5.367±0.167	6.667±0.367	2.567±0.200	3.133±0.233	ND
Isorhamnetin-3-O-rutinoside	ND	ND	ND	ND	ND	5.267±0.100	6.167±0.200	0.933±0.067	1.233±0.100	8.533±0.400
Syringetin-3-O-galactoside	36.567±1.133	38.533±2.500	22.333±1.600	22.133±0.767	27.967±1.233	40.000±1.267	47.700±1.100	30.200±2.233	40.967±1.833	14.933±1.133
Myricetin-3-O-galactoside	43.600±3.800	42.733±1.633	41.167±3.333	37.200±2.367	51.700±1.967	18.867±1.167	12.000±0.600	ND	2.067±0.167	29.500±2.700

Myricetin-3-O-glucoside	58.800±3.533	55.433±3.100	44.600±4.200	41.067±3.333	52.833±4.500	ND	ND	ND	ND	ND
Gallic acid	11.400±0.800	16.900±0.533	5.100±0.333	6.767±0.167	15.800±0.633	1.767±0.067	1.433±0.067	0.400±0.000	0.567±0.000	2.967±0.233
Protocatechuic acid	14.700±0.700	20.700±1.633	6.700±0.400	8.767±0.367	38.200±1.467	2.333±0.133	2.100±0.100	1.800±0.133	1.367±0.100	1.467±0.033
Gallocatechin	16.833±1.167	19.667±1.567	28.300±1.900	25.200±0.600	18.767±0.533	3.133±0.167	2.367±0.100	ND	ND	0.900±0.033
Procyanidin B1	17.033±0.500	21.567±1.200	8.500±0.333	7.833±0.333	6.233±0.333	151.433±7.867	124.400±6.233	184.600±12.933	174.500±6.100	15.133±0.767
Procyanidin B4	9.600±0.267	12.567±0.767	11.500±0.400	13.567±0.600	11.967±0.633	ND	ND	ND	ND	ND
Procyanidin B2	121.000±8.100	137.967±9.933	109.567±8.533	109.900±7.800	91.233±5.467	19.267±0.433	15.867±0.433	12.967±0.467	11.933±0.767	4.900±0.300
Catechin	21.100±1.133	25.800±1.033	25.100±0.633	23.833±0.733	11.000±0.367	96.933±4.367	91.867±3.133	157.767±3.167	179.233±11.467	10.233±0.333
Vanillic acid	ND	ND	ND	ND	ND	1.900±0.100	2.033±0.133	ND	ND	ND
Chlorogenic acid	568.633±39.233	614.000±25.800	795.933±27.067	787.400±23.633	732.633±50.567	1205.333±77.133	1034.600±30.000	1509.067±78.467	1462.500±43.867	1119.967±33.600
Caffeic acid	5.067±0.333	5.367±0.333	2.867±0.167	2.700±0.100	3.200±0.200	48.633±0.967	24.533±0.967	4.833±0.133	4.400±0.100	3.333±0.267
4-O-Caffeoylquinic acid	ND	ND	ND	ND	ND	7.033±0.533	6.567±0.267	8.467±0.600	9.867±0.300	16.600±0.767
Syringic acid	2.600±0.100	3.500±0.233	1.300±0.067	1.933±0.133	2.767±0.167	8.500±0.667	11.400±0.767	6.067±0.433	7.633±0.500	8.233±0.567
Epicatechin	50.433±3.933	58.933±1.700	96.300±2.500	94.800±5.133	53.867±1.833	10.667±0.233	9.267±0.467	9.967±0.500	9.700±0.567	3.067±0.233
p-Coumaric acid	3.233±0.167	3.733±0.067	0.700±0.067	0.767±0.033	1.800±0.100	ND	ND	ND	ND	ND
Procyanidin C1	66.533±2.000	76.367±5.267	48.167±2.500	49.400±3.967	38.833±2.967	8.267±0.633	7.600±0.433	6.833±0.200	6.667±0.500	4.467±0.100
Ferulic acid	ND	ND	ND	ND	ND	5.067±0.367	4.900±0.100	ND	ND	ND
Procyanidin A2	4.633±0.267	5.633±0.200	2.067±0.133	2.700±0.167	2.700±0.133	ND	ND	ND	ND	ND
Hyperoside	322.500±23.867	362.533±29.000	241.367±8.933	252.700±17.933	488.433±35.167	367.467±24.633	467.633±34.600	217.633±11.767	315.667±14.200	200.800±11.433
Isoquercitrin	72.600±1.800	83.300±2.000	52.500±1.633	51.067±2.867	59.000±1.667	64.967±4.433	82.500±3.867	106.733±7.267	148.733±8.333	93.100±5.033
Quercetin-3-O-robinobioside	ND	ND	ND	ND	ND	1.733±0.067	2.200±0.067	4.800±0.333	6.933±0.467	1.167±0.100
Rutin	ND	ND	ND	ND	ND	14.300±0.400	16.967±0.700	38.033±2.067	50.600±1.967	97.700±7.033
Myricetin	4.200±0.133	3.333±0.233	5.467±0.233	4.300±0.267	8.867±0.633	ND	ND	ND	ND	ND
Quercitrin	32.533±1.900	38.433±2.333	15.233±0.467	16.967±0.600	29.100±1.633	ND	ND	ND	ND	455.133±32.767
Quercetin-3-O-glucose-6-acetate	ND	ND	ND	ND	ND	0.900±0.033	1.200±0.067	119.400±5.000	173.600±12.833	3.133±0.133
Kaempferol-3-O-glucoside	ND	ND	ND	ND	ND	2.900±0.167	3.933±0.200	6.767±0.433	9.567±0.433	7.300±0.167

Abbreviations: VM, *Vaccinium myrtillus* (wild bilberry); VC, *Vaccinium corymbosum* (cultivated blueberry); A, acidic extraction conditions (pH 4); N, neutral extraction conditions (pH 7); DW, dry weight; ND, not detected.
